# Multi-symptom asthma as an indication of disease severity in epidemiology

**DOI:** 10.1186/2045-7022-3-S1-P6

**Published:** 2013-05-03

**Authors:** Linda Ekerljung, Apostolos Bossios, Jan Lötvall, Anna-Carin Olin, Eva Rönmark, Göran Wennergren, Kjell Torén, Bo Lundbäck

**Affiliations:** 1 University of Gothenburg, Sweden; 2 University of Gothenburg, Institute of Medicine/Krefting Research Centre, Sweden; 3 University of Gothenburg, Institute of Medicine/Dept of Occup & Envir Med, Sweden; 4 University of Umeå, Dept of Public Health & Clinical Medicine, Sweden; 5 University of Gothenburg, Dept of Pediatrics, Sweden

## 

Epidemiological questionnaires have failed to identify individuals with severe asthma. The extent of symptoms of asthma can, however, easily be established in epidemiological studies by identification of multiple symptoms. We hypothesized that reporting of multiple symptoms of asthma reflects uncontrolled disease and can be a sign of more severe asthma. The aims of the current study were, therefore, to determine the prevalence and determinants of multi-symptom asthma. In this paper we report our definition of multi-symptom asthma and its clinical characteristics. A postal questionnaire was mailed to 30,000 randomly selected subjects aged 16**–**75 yrs. A subgroup underwent detailed clinical examinations including lung function test, exhaled NO, methacholine test in addition to a detailed clinical history by using structured interview. Multi-symptom asthma was defined as questionnaire reported physician-diagnosed asthma, use of asthma medication, recurrent wheeze, attacks of shortness of breath, and at least one additional respiratory symptom. The overall prevalence of physician-diagnosed asthma was 8.3%, while of multi-symptom asthma the prevalence was 2.0% (women 2.4%, men 1.5%, p < 0.001). Multi-symptom asthma versus other asthma was associated with lower FEV1 (88.8% pred vs. 98.8% pred), higher FeNO (29.3 ppb vs. 23.2 ppb), a greater proportion having PD20 < 1.96 mg methacholine chloride (82.9% vs. 58.7%), all statistically highly significant. The same pattern was found for asthma exacerbations, emergency department visits and hospitalizations. All respiratory symptoms were more common in multi-symptom asthma compared with other asthma, and that was true also for symptoms of bronchitis, rhinitis and rhino-sinusitis. In contrast, allergic rhinitis and allergic sensitization were not more common in multi-symptoms asthma than in other asthma. Multi-symptom asthma cannot be used for defining severe asthma. We conclude, however, that multi-symptom asthma, as we defined the condition, is related to signs of more severe disease and could be used as an epidemiological marker of asthma severity.

